# Arsenic Exposure and Cancer-Related Proteins in Urine of Indigenous Bolivian Women

**DOI:** 10.3389/fpubh.2020.605123

**Published:** 2020-12-14

**Authors:** Jessica De Loma, Anda R. Gliga, Michael Levi, Franz Ascui, Jacques Gardon, Noemi Tirado, Karin Broberg

**Affiliations:** ^1^Institute of Environmental Medicine, Karolinska Institutet, Stockholm, Sweden; ^2^Programa de Salud Familiar Comunitaria e Intercultural, Ministerio de Salud Bolivia, La Paz, Bolivia; ^3^Hydrosciences Montpellier, Université de Montpellier, Institut de Recherche pour le Développement, Centre National de la Recherche Scientifique, Montpellier, France; ^4^Genetics Institute, Universidad Mayor de San Andrés, La Paz, Bolivia

**Keywords:** arsenic, Andes, Bolivia, FASLG, TFPI2, biomarker, carcinogenic

## Abstract

Indigenous people living in the Bolivian Andes are exposed through their drinking water to inorganic arsenic, a potent carcinogen. However, the health consequences of arsenic exposure in this region are unknown. The aim of this study was to evaluate associations between arsenic exposure and changes in cancer-related proteins in indigenous women (*n* = 176) from communities around the Andean Lake Poopó, Bolivia. Arsenic exposure was assessed in whole blood (B-As) and urine (as the sum of arsenic metabolites, U-As) by inductively coupled plasma-mass spectrometry (ICP-MS). Cancer-related proteins (*N* = 92) were measured in urine using the proximity extension assay. The median B-As concentration was 2.1 (range 0.60–9.1) ng/g, and U-As concentration was 67 (12–399) μg/L. Using linear regression models adjusted for age, urinary osmolality, and urinary leukocytes, we identified associations between B-As and four putative cancer-related proteins: FASLG, SEZ6L, LYPD3, and TFPI2. Increasing B-As concentrations were associated with lower protein expression of SEZ6L, LYPD3, and TFPI2, and with higher expression of FASLG in urine (no association was statistically significant after correcting for multiple comparisons). The associations were similar across groups with different arsenic metabolism efficiency, a susceptibility factor for arsenic toxicity. In conclusion, arsenic exposure in this region was associated with changes in the expression of some cancer-related proteins in urine. Future research is warranted to understand if these proteins could serve as valid biomarkers for arsenic-related toxicity.

## Introduction

Inorganic arsenic (iAs) is classified as a class I human carcinogen (1). Chronic exposure to iAs has been associated with multiple types of cancer, including cancers of the skin, lung, bladder, kidney, liver, and prostate (1–4). Exposure to iAs has also been associated with non-carcinogenic health effects such as cardiovascular disease, diabetes, and immunotoxicity (4–6).

Leakage of iAs from volcanic bedrocks into groundwater used as drinking water is one of the main contributors to human exposure, especially in Latin America where more than 14 countries present elevated iAs in drinking water (7). Arsenic-related cancer has been evaluated in some Latin American countries, mainly Chile, Argentina, and Mexico (6). In the Chilean city of Antofagasta and in the Córdoba province of Argentina, studies have consistently found an increased lung, bladder, and kidney cancer mortality in relation to chronic exposure to iAs (8–10). Recently, iAs was linked to laryngeal cancer mortality in Chile (11), and chronic exposure to iAs and risk for breast cancer was evaluated in Latin America (12–14). In the Bolivian Andes, we have shown that women living around Lake Poopó are exposed to iAs (15). However, very little is known about cancer or any toxicity in relation to iAs exposure in Bolivia.

A growing body of knowledge supports the use of urinary proteins to detect early signs of cancer and other diseases (16–19). Although several epidemiological studies have investigated protein biomarkers associated with iAs in humans (20–22), the relation between urinary proteins and arsenic remains unexplored. To our knowledge, only one study has previously explored urinary proteins in relation to iAs exposure. In that study, limited to proteins within 2–10 kDa of size, highly exposed individuals (>100 μg/L U-As) from the United States and Chile had decreased expression of human beta-defensin 1 (HBD-1), an antimicrobial protein and potential tumor suppressor gene in urological cancers (23). The effect of iAs on urinary proteins has been overlooked in the scientific literature despite its potential for biomarker discovery. Therefore, the aim of this cross-sectional study was to evaluate the associations between putative cancer-related proteins in urine and arsenic exposure in indigenous individuals from the Bolivian Andes.

## Materials and Methods

### Study Participants and Sample Collection

We recruited women living in villages around Lake Poopó, located in the southern region of the Bolivian highlands, at 3,686 m above sea level (15). This area is characterized by arsenic-rich soils, common in the Andean regions. Recruitment of 201 women took place during five field trips organized between September 2015 and November 2017 from a total of 10 villages around Lake Poopó. Women with no major health conditions were recruited on a voluntary basis (one woman with a brain tumor and tuberculosis was excluded). We recruited women from two ethnic groups, Aymara-Quechua and Uru, that inhabit this region. During recruitment, the women had similar eating habits and lifestyles in all villages, based on questionnaire data. Men were not recruited into the study because they usually worked away from their village leading to a different arsenic exposure. For this study, we started from a subset of women (*n* = 182) for which we had most complete biological data, including genetic data which is not presented in this study. Out of these women, we excluded five that presented extreme levels of nitrites in urine (based on urine strip determination described further on), and one woman without urine strip data. In total, 176 women were included in the current study, of which 149 identified themselves as Aymara-Quechua and 27 as Uru. This study was approved by the Comité Nacional de Bioética (Bolivia) and the Regional Ethic Committee of Karolinska Institutet (Sweden). Prior to recruitment and sampling, participants were informed orally and in written form about the project, and signed informed consent was obtained.

We carried out personal interviews including information such as age, ethnicity, weight, height, blood pressure (measured in sitting position), health status, and frequency of chewing coca leaves (15). Body mass index was not used since Andean populations are known to have higher values as a consequence of living at high altitude without them being associated with excess body fat (15, 24, 25). Spot urine samples were collected throughout the day during the field trips. The women were given instructions on how to collect urine samples, including wet wipe cleaning and mid-stream urine collection to minimize contamination. The urine samples were collected in 20 mL polyethylene bottles, previously confirmed as free of trace elements. Venous blood samples were obtained with BD Vacutainer Eclipse blood collection needles (Becton Dickinson, USA) in Trace Elements NH Sodium Heparin tubes (Vacuette, Greiner Bio, Austria), or in Lithium Heparin tubes (Vacuette) when the Sodium Heparin tubes were not available. Due to field trip limitations, it was not possible to separate plasma. Urine and blood samples were stored directly after sampling at −18°C in a portable freezer (ARB, Australia), and stored at Universidad Mayor de San Andrés (La Paz, Bolivia) at −20°C until further shipment. Samples were transported on dry ice to Karolinska Institutet (Stockholm, Sweden), where they were stored at −20°C for long-term storage. All urine and blood samples had similar freeze-thaw cycles.

### Protein Measurements in Urine

Urine samples for protein measurements were thawed overnight at 4°C. After thoroughly mixing each sample, it was aliquoted, debris were separated by centrifugation (845 g for 10 min), and 50 μL of the supernatant was added to 96-well plates (Thermo Fisher Scientific, USA) covered with MicroAmp Clear Adhesive Film (Thermo Fisher Scientific). Samples were transported on ice from Karolinska Institutet (Stockholm, Sweden) to Olink Proteomics (Uppsala, Sweden).

Urine samples were analyzed with the Proseek Multiplex Oncology II panel (*N* = 92 proteins) based on the Proximity Extension Assay (PEA) developed and performed by Olink Proteomics. The PEA is a dual-recognition immunoassay which uses a pair of antibodies labeled with DNA oligonucleotides specific for each pre-determined protein on the panel. When both labeled antibodies bind to the target protein, the oligonucleotide labels are in enough proximity to hybridize and create a PCR target sequence that will be quantified by real-time PCR. The Oncology II panel includes proteins belonging to biological process ontologies relevant to cancer development: angiogenesis (*n* = 20), apoptotic process (*n* = 34), cell adhesion (*n* = 35), cell differentiation (*n* = 42), cell motility (*n* = 30), cell proliferation (*n* = 43), cellular metabolic process (*n* = 47), cellular response to stress (*n* = 23), chemotaxis (*n* = 14), extracellular matrix organization (*n* = 9), immune response (*n* = 27), MAPK cascade (*n* = 25), proteolysis (*n* = 19), response to hypoxia (*n* = 3), and other gene ontology terms (*n* = 9). Methodological details, data processing, quality control and normalization are described by Assarsson et al. (26) and are available online at https://www.olink.com. Protein level data obtained by the Olink Proteomic facility are presented as Normalized Protein eXpression (NPX) values, which have arbitrary units on a log2-scale. The intra-assay coefficient of variance (CV%) was below 20% for all proteins (only one protein above 15%), while the inter-assay CV% was below 30% for all proteins (only two proteins between 20 and 30%). The limit of detection (LOD) for each protein assay is defined as three times the standard deviation above background level based on internal controls. For downstream analyses, we included proteins that had more than 40% of the samples above the LOD, i.e., 45 proteins. Since this multiplex assay was initially developed to detect proteins in plasma, we expected that some of the selected proteins in the panel would not be present to the same extent in urine. Therefore, we were less stringent with the LOD cut-off compared to other studies that included proteins measured in plasma with more than 80–90% of the samples above LOD (27, 28).

### Arsenic Exposure and Metabolism Efficiency Assessment

For this study, we used arsenic concentrations in whole blood (B-As), and not in urine, as a biomarker of exposure in order to avoid the potential co-excretion of arsenic and proteins in urine (29).

We previously described that the women in this region had elevated concentrations of arsenic and lithium in urine, and that arsenic and lithium concentrations in urine were correlated (r_S_ = 0.47, *p*-value < 0.001) (15). Therefore, in this study we also used lithium concentrations in whole blood (B-Li; correlation with B-As: r_S_ = 0.44, *p*-value < 0.001) to evaluate potential confounding.

The blood samples were prepared for inductively coupled plasma-mass spectrometry (ICP-MS; operating conditions in Supplementary Table 1) by a direct alkali dilution method (30). Briefly, blood samples were diluted 1:17–44 with an alkali solution consisting of 2% butanol (Honeywell Research Chemicals, Germany), 0.05% EDTA (Sigma-Aldrich, USA), 0.05% Triton X-100 (Sigma-Aldrich), 1% NH_4_OH (Romil, UK), and 20 μg/g internal standards ^45^Sc, ^72^Ge, ^103^Rh, ^175^Lu, and ^193^Ir. Before analysis, the diluted samples were sonicated for 5 min, and centrifuged at 694 g for 5 min. The Agilent 7900 ICP-MS (Agilent Technologies, Japan) equipped with an octopole reaction system (ORS) collision/reaction cell technology was used for measuring concentrations of arsenic and lithium. The LOD for each element was determined as three times the standard deviation of analyzed blanks (alkali solution) and as signal/noise = 3. The limit of quantification (LOQ) was determined as 10 times the standard deviation of analyzed blanks. The analysis precision was estimated by measuring two in-house blood samples in triplicate. As quality control, two commercially available whole blood reference materials were analyzed: Seronorm™ Trace Elements Whole Blood L-1 (LOT 1702821) and L-2 (LOT 1702825), and the obtained average arsenic values (1.9 ± 0.1 μg/kg and 10 ± 1 μg/kg) were in agreement with the reference values (2 ± 0.4 μg/kg and 11.6 ± 2.4 μg/kg). Certified values were converted from μg/L to μg/kg by dividing by the average density of blood (1.055 kg/L). In addition, the reference materials were spiked with 2–1385 μg/kg lithium, and on average 101% of the added lithium was recovered. Blanks and reference materials were treated together with the collected whole blood samples and analyzed in the beginning, in the middle, and at the end of each analysis. For some individuals, blood samples for element analysis were collected in Lithium Heparin tubes instead of Trace Elements NH Sodium Heparin tubes (Vacuette, Greiner Bio), and therefore B-Li results could not be obtained for all individuals. Leach tests for both types of Vacuette tubes were performed, and no traces of arsenic were detected in neither of them (data not shown).

In the human body, iAs is metabolized via the one-carbon cycle by reducing As(V) to As(III), and methylating As(III) into methylarsonic acid (MMA) and further into dimethylarsinic acid (DMA). The metabolism is not complete, and all four arsenic species are to varying degrees excreted in urine (31). We previously measured arsenic in urine, both as total arsenic in urine (including organic forms such as arsenobetaine from seafood) and as the sum of iAs metabolite concentrations (iAs + MMA + DMA; U-As) in urine, and concluded that this study group is mainly exposed to inorganic forms (15).

### Covariates

Urine test strips (Combur-7 Test strips, Roche, Switzerland) were used immediately after sample collection to determine urinary pH, glucose, ketones, leukocytes, nitrites, proteins, erythrocytes, and hemoglobin. These urine reagent strips are commonly used in clinical settings to detect diabetes and other kidney and urinary tract diseases in a semiquantitative manner. Results were obtained and graded on a discrete numerical scale (0, 1, 2, 3, or 4) by comparing the color of the patches on the strip with the colors on the label, according to the manufacturers indications.

To account for variations in urine dilution, we previously measured specific gravity and osmolality (15). In this study, we only included osmolality since it is strongly correlated with specific gravity in the study group (r_S_ = 0.99, *p-*value < 0.001), and because specific gravity can be influenced by the presence of proteins and glucose (32).

We evaluated if differences in storage time affected the relative protein concentrations by including the field trip as a covariate (~26 months between the first and the last sampling occasion).

We also assessed if the association between B-As and cancer-related proteins was influenced by the efficiency of arsenic metabolism, determined as the relative concentration (%) of the different iAs metabolites in urine. Higher fractions of iAs and MMA in urine are associated with higher risk of adverse health outcomes, and therefore a low efficiency of arsenic methylation is considered a susceptibility factor for arsenic toxicity (2, 33). We stratified the study group as below and equal, or above median %MMA.

### Statistical Analyses

All analyses were performed with RStudio (version 1.1.423) using R (version 3.6.2). General characteristics of the study group that are continuous variables and protein levels as NPX values are presented as median and interquartile range (IQR), while categorical variables are presented as percentage or number of individuals per category. NPX values below LOD were excluded from the dataset.

Linear models of principal components were analyzed to assess the influence of different covariates on the overall protein variation. For this, we used the *prince* and *prince*.*plot* functions within the *swamp* package. These functions perform a principal component analysis for the overall protein expression variation and then perform a linear regression between each variable and principal component to evaluate their association. The heat map presents -log_10_(*p-*value) for the associations, and variables were hierarchically clustered using the *hclust* function. Since the *prince* function does not handle missing data, and imputation was not optimal due to limited input data, we used the NPX dataset substituting values below the LOD by the LOD value specific for each protein. Spearman correlation-tests were carried out between the covariates that were statistically significant in the linear model of principal component analyses. Covariates that were significantly associated with at least one of the 10 principal components and not correlated between each other, as assessed by Spearman correlation-test, were further included in linear regression models used to evaluate individual protein variation and to assess the relation between these proteins and B-As (see below).

To evaluate how much the selected covariates explained the variation of each protein, univariate linear regression analyses were performed. Each model included one protein (as dependent variable) and one covariate (as independent variable) at a time. The variance explained (R^2^) was presented as stacked bar plots. To assess the association between B-As and protein expression, multivariable-adjusted linear analyses were performed, adjusting for the covariates that were significantly associated with the overall protein variation. Standardized beta coefficients were obtained for all covariates in the model and presented in stacked bar plots. One individual was excluded from these analyses since no B-As data was available. Similar analyses were performed also adjusting for B-Li as sensitivity analyses. The top associated proteins with B-As were further evaluated with Spearman pairwise correlations, with scatter plots against B-As including a stratification by arsenic metabolism efficiency, and with boxplots to compare between low and high exposed participants. The categorization between low and high exposed was done by splitting by the median B-As. Since the urinary proteins were not normally distributed, we performed Wilcoxon-tests to evaluate the differences between groups.

## Results

### Characteristics of the Study Participants

Information on the women's anthropometric and lifestyle characteristics, urinary characteristics, and exposure biomarkers are included in Table 1. The exposure to iAs from drinking water of the current study group (U-As median 67 μg/L, range 12–399 μg/L; B-As median 2.1 ng/g, range 0.60–9.1 ng/g) is comparable to that of the whole study population (*N* = 201 women, U-As median 65 μg/L, range 12–407 μg/L) (15). Furthermore, U-As (as sum of metabolites, adjusted for average osmolality) and B-As were strongly correlated (r_S_ = 0.85, *p*-value < 0.001; Supplementary Figure 1) reflecting that both matrices are reliable biomarkers for iAs exposure in this study group. Only two women reported consuming alcoholic beverages frequently (every other week), and seven reported smoking tobacco (yes/no). Based on the urine strips, no woman had ketones, two had elevated glucose, 10 nitrites, 16 proteins, 40 hemoglobin, and 12 erythrocytes in urine.

**Table 1:** Characteristics of the individuals included in the study (*n* = 176).

	***n***	**Median (IQR)^a^**
**General characteristics**		
Age (years)	176	36 (28–48)
Weight (kg)	176	60 (53–70)
Height (cm)	176	149 (146–153)
Coca chewing (yes)^b^	174	74%
Systolic blood pressure (mmHg)	175	100 (90–110)
Diastolic blood pressure (mmHg)	175	70 (60–70)
**Urine characteristics**		
Specific gravity	176	1.02 (1.01–1.02)
Osmolality (mOsm/kg)	176	764 (564–883)
Urine pH	176	5.5 (5.0–6.1)
Urine leukocytes (0/1/2/3)^c^	176	146/10/17/3
**Exposures**		
U-As (μg/L)^d^	176	67 (47–111)
iAs (%)	176	12 (8.6–17)
MMA (%)	176	7.7 (6.2–9.6)
DMA (%)	176	79 (74–84)
B-As (ng/g)^e^	175	2.1 (1.4–3.0)
B-Li (ng/g)^e^	159	18 (9.4–30)

a *Data presented as median and interquartile range (IQR) unless otherwise stated*.

b *Percentage of individuals who reported chewing coca leaves regularly*.

c *Number of individuals in each category of urine leukocyte levels, classified according to the urine strips Combur 7 Test*.

d *Sum of urinary iAs metabolites adjusted for average urinary osmolality (727 mOsm/kg) of the total study population as described in De Loma et al. (15)*.

e *Measured in whole blood*.

### Covariates Explaining Protein Variation in Urine

Descriptive statistics of the 92 cancer-related proteins measured by the Multiplex Oncology II panel are shown in Supplementary Table 2. Out of the proteins initially measured, 45 proteins were further evaluated, i.e. those with > 40% of observations above LOD. To assess to which extent characteristics of the study participants were associated with variation of these proteins, we performed linear regression models of principal components (Supplementary Figure 2). Age, urinary osmolality, urinary leukocytes and urinary pH significantly explained the overall protein variation in urine. Urinary pH and osmolality were correlated (r_S_ = −0.21, *p*-value = 0.005), while none of the other covariates presented pairwise correlations, and therefore urinary pH was not included in further analyses. No significant associations were found for coca chewing, ethnicity, or sampling field trip. None of the other markers from the urine strips (glucose, nitrites, proteins, hemoglobin, nor erythrocytes) were associated with the overall protein variation of the cancer-related proteins. In addition, there were no differences in protein expression between ethnicities (data not shown).

**Figure 1:**
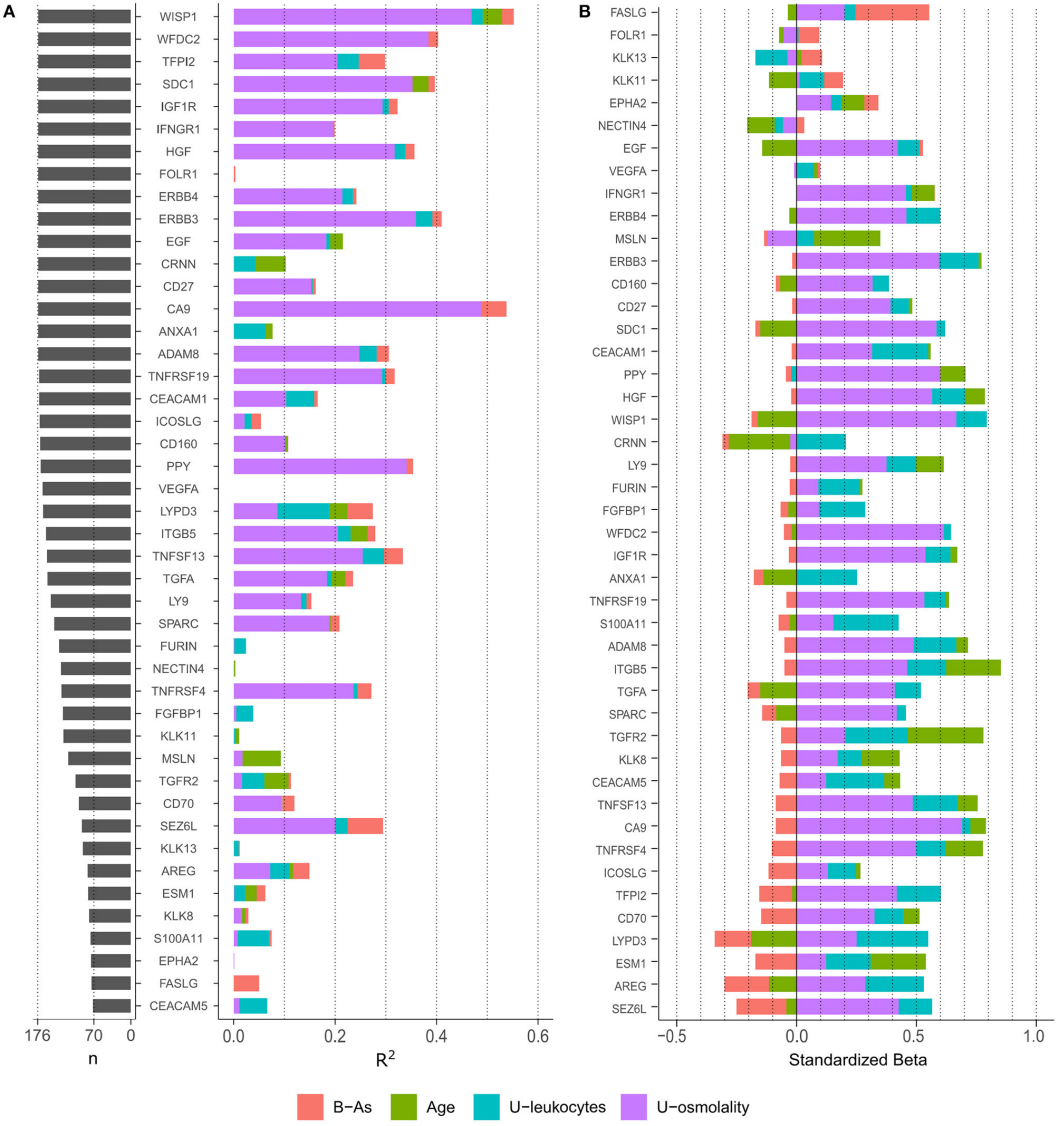
**(A)** Number of observations (*n*) for each protein and variance explain (R^2^) by each variable in independent linear regression models, including one independent variable in the model at a time. Only proteins with > 40% of observations above LOD were included. **(B)** Standardized effect of each variable on protein levels. The plot depicts the standardized beta coefficient of each variable (as independent variables) in the multivariable-adjusted linear regression model including the protein as dependent variable. Proteins in the plot are ordered by the effect of B-As on protein levels (B coefficient for B-As).

To evaluate the protein variance explained by each covariate independently, we performed linear regression analyses including each covariate individually (age, urinary osmolality, urinary leukocytes, and B-As as the exposure of interest; Figure 1A). Urinary osmolality explained the variation of most proteins measured in urine, in some cases explaining up to 49% of the variation. However, 13 proteins were not influenced by osmolality (R^2^ <1%: FOLR1, CRNN, ANXA1, VEGFA, FURIN, NECTIN4, FGFBP1, KLK11, KLK13, ESM1, S100A11, EPHA2, and FASLG). Age explained the variation (R^2^: 1.3–7.5%) of 11 proteins: WISP1, SCD1, EGF, CRNN, ANXA1, LYPD3, ITGB5, TGFA, MSLN, TGFR2, and ESM1. The only marker selected from the urine strip test was urinary leukocytes, as a proxy for inflammation of the urinary tract, which explained between 1 and 10% of the variation of 23 proteins of which LYPD3, ANXA1, and S100A11 showed the highest R^2^ (10, 6.3, and 6.2% respectively). B-As explained 5–7% of the protein variation of TFPI2, LYPD3, SEZ6L, and FASLG.

### Urinary Proteins Associated With Arsenic Exposure

**Figure 2:**
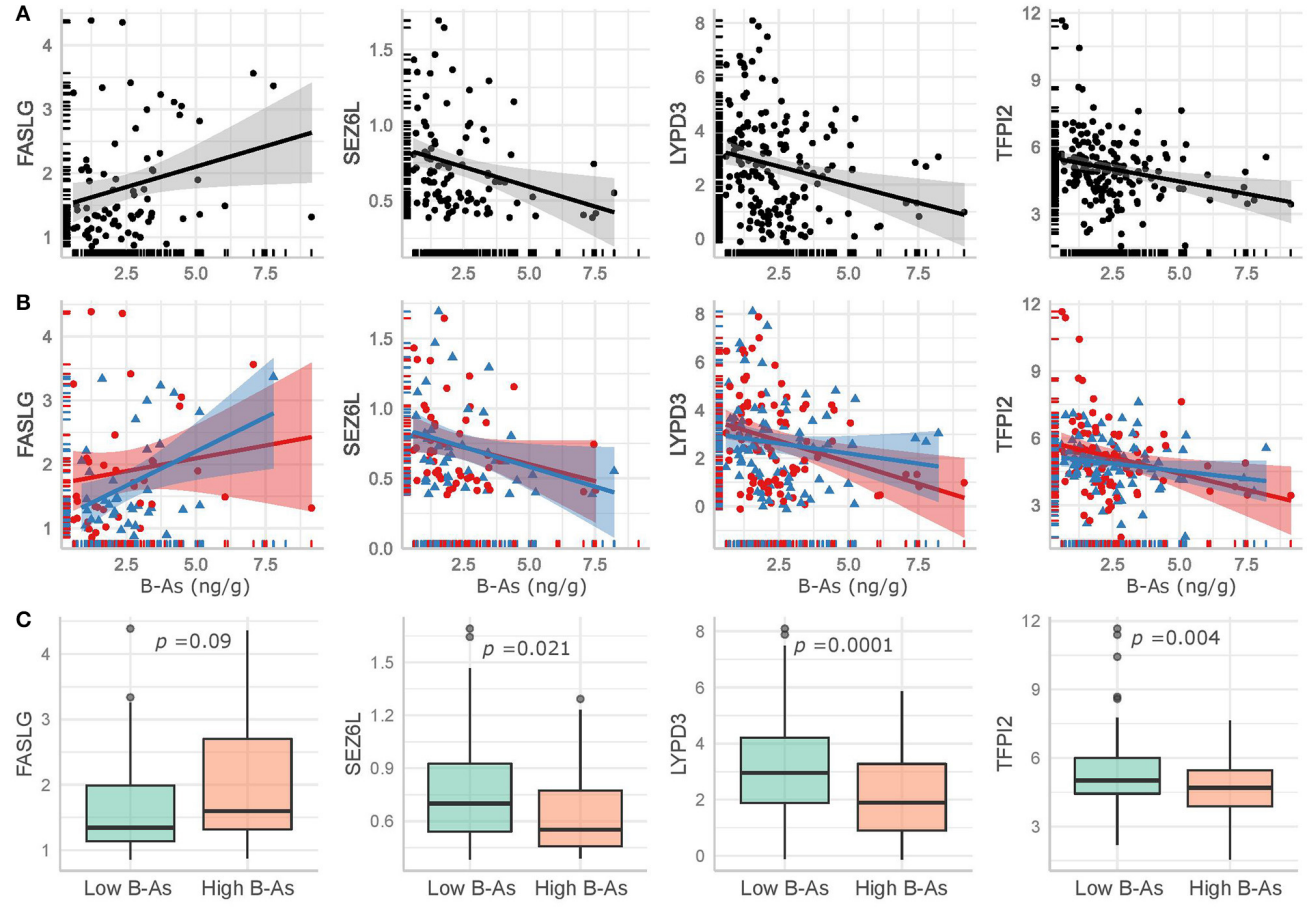
Top proteins in urine significantly associated (*p*-value < 0.05) with B-As in the adjusted linear regression models, including non-adjusted linear regression estimates and their confidence intervals for **(A)** the total study group and **(B)** stratified by arsenic methylation efficiency, as below (red circles and line) or above (blue triangles and line) the median %MMA. **(C)** Comparison of the top proteins in urine between low and high exposed individuals, divided by the median B-As. The *p*-values for Wilcoxon-tests are included.

We further examined how B-As was associated to cancer-related proteins in urine by multivariable linear regression models adjusted for age, urinary leukocytes, and urinary osmolality. Out of the 45 proteins, four were associated (*p-*value < 0.05) with B-As: Tumor necrosis factor ligand superfamily member 6, FASLG; Seizure 6-like protein, SEZ6L; Ly6/PLAUR domain-containing protein 3, LYPD3; and Tissue factor pathway inhibitor 2, TFPI2 (Figure 1B, Table 2, and Supplementary Table 3). None of the associations were statistically significant after adjusting for multiple testing. The relative protein expression of SEZ6L, LYPD3, and TFPI2 decreased with higher B-As concentrations, while FASLG increased with increasing B-As concentrations (Table 2 and Figure 2A). Differences in arsenic metabolism efficiency did not change the association between these top proteins and B-As (Figure 2B). In addition, we explored if these top proteins were significantly different between individuals with low or high arsenic exposure, by splitting by median B-As (Figure 2C). Individuals highly exposed to arsenic had significantly lower SEZ6L, LYPD3, and TFPI2 levels (*p*-value = 0.021, 0.0001, 0.004, respectively). Highly exposed individuals had higher levels of FASLG, although not significantly (*p*-value = 0.09).

**Table 2:** Top 10 proteins in urine associated with B-As.

**Main analysis^a^**				
**Protein**	***n***	**R**^**2**^ **(%)**	**B (95% CI)**	***p*****-value**
FASLG	74	5	0.155 (0.034, 0.276)	0.013
SEZ6L	93	25	−0.038 (−0.072, −0.004)	0.028
LYPD3	166	23	−0.172 (−0.331, −0.014)	0.034
TFPI2	175	24	−0.125 (−0.249, −0.001)	0.049
AREG	82	15	−0.096 (−0.204, 0.012)	0.080
CA9	175	49	−0.05 (−0.112, 0.013)	0.119
ESM1	81	7	−0.067 (−0.152, 0.019)	0.124
ICOSLG	172	3	−0.097 (−0.225, 0.03)	0.134
CD70	98	11	−0.046 (−0.106, 0.015)	0.137
TNFRSF4	131	27	−0.066 (−0.164, 0.032)	0.183

*Data presented as B coefficients and 95% confidence intervals (CI) for the association with B-As*.

*No proteins passed the Bonferroni adjustment for multiple comparisons (p-value threshold = 0.05/45 = 0.001)*.

a *Multivariate linear regression models adjusted for age, urinary osmolality, and urinary leukocytes*.

We also explored the correlation between the top proteins that were associated with B-As. Spearman pairwise correlations between SEZ6L, LYPD3, and TFPI2 were observed, but not for FASLG (Supplementary Figure 3). We considered performing downstream analyses, e.g., pathway enrichment, but the number of proteins associated with B-As was not enough for such analyses to be suitable. Sensitivity analyses were performed including B-Li in the models. The effect estimates for the associations between B-As and FASLG, SEZ6L, LYPD3, and TFPI2 did not change more than 10%, although they were no longer significant (*p-*value < 0.05), probably due to the decrease in sample size (Table 2 and Supplementary Table 3).

## Discussion

To our knowledge, this study is the first to evaluate the toxicity of environmental iAs exposure in individuals from Bolivia. We identified four proteins (FASLG, SEZ6L, LYPD3, and TFPI2) measured in urine that were associated with B-As in indigenous women from the Bolivian Andes. Arsenic concentrations in blood were positively associated with relative protein expression of FASLG and inversely associated with SEZ6L, LYPD3, and TFPI2. These associations were not statistically significant when correcting for multiple comparisons likely due to limited sample size. When comparing individuals with high vs. low exposure to arsenic, SEZ6L, LYPD3, and TFPI2 were significantly decreased in the high exposure group, while FASLG was non-significantly increased. The individual proteins are discussed below including previous relations reported for arsenic, when known. So far, there is no knowledge about whether urinary levels of these proteins are predictive of cancer. Longitudinal research is needed to establish whether these proteins serve as early biomarkers of arsenic-related carcinogenicity, or if these associations are a consequence of an adaptive response to the exposure. This study also identified other influential factors for the variation in protein expression in urine, such as urinary dilution and age, which is important information for future use of these proteins as potential risk biomarkers.

FASLG binds to Fas receptors and regulates an apoptotic signaling pathway (34, 35). FASLG has a dual function; it both promotes and inhibits cell death depending on the cell type (36). Elevated serum concentrations of soluble FASLG have been detected in patients with leukemia, lymphoma and multiple solid tumors (37). Arsenic has been linked to FASLG protein expression, mainly in *in vitro* studies of the cancer drug arsenic trioxide, which is currently used for treatment of acute promyelocytic leukemia. Different leukemia cell lines exposed to arsenic trioxide showed increased gene or protein expression of FASLG (38–40). Human studies of environmental exposure to iAs and FASLG are few and inconclusive. Women highly exposed to iAs (median U-As 276 μg/L) from the Argentinean Andes presented higher *FASLG* gene expression in sorted T-cells compared to women with lower exposure (median U-As 65 μg/L) (41). In contrast, five individuals from Mexico exposed to iAs (median 224.3 mg/g creatinine) showed lower gene expression of *FASLG* in peripheral blood mononuclear cells (including T cells, B cells, and NK cells) compared to five unexposed individuals (iAs < 2.2 mg/g creatinine) (42).

TFPI2 is an inhibitor of the tissue factor pathway involved in blood coagulation. Gene expression of *TFPI2* was decreased in samples of bladder tumors (43) and cervical tumors (44), while TFPI2 protein concentrations in serum were higher in ovarian clear cell adenocarcinoma patients (45, 46). Hypermethylation, generally associated with decreased gene expression (47), was detected for *TFPI2* in multiple types of cancer compared to normal adjacent tissues (48–52). A human prostate epithelial cell line transformed by chronic exposure to arsenite presented decreased *TFPI2* gene expression (53), in line with our findings. On the contrary, normal human lung cells exposed to sodium arsenite for 30–60 days showed an upregulation of *TFPI2* gene expression (54).

Two of the proteins identified, SEZ6L and LYPD3, have to our knowledge not been related to arsenic before. SEZ6L is a transmembrane protein in the endoplasmic reticulum and the cell membrane whose function is still unclear. In our study, lower urinary expression of SEZ6L was associated with increasing arsenic exposure. Interestingly, deletions in the *SEZ6L* gene have been found in primary lung tumor cells (55), and loss-of-function variants in *SEZ6L* have been associated with increased risk of lung cancer (56). *SEZ6L* is also reported as a fusion gene in some lung cancers, melanoma and skin cancer (57).

LYPD3 is a glycosylphosphatidylinositol-anchored urokinase receptor involved in cell-matrix interactions and metastasis (58, 59). In contrast to our findings of lower relative protein expression in relation arsenic exposure, enhanced protein expression of LYPD3 has been identified in tissues from urothelial cancers (59), breast cancers (60), melanoma (61), and lung cancers (62). Recently, high protein expression levels of LYPD3 in tumor tissues have been recognized as a biomarker of poor prognosis for lung cancer patients (63, 64).

We also identified factors that explained the overall variation of the 45 proteins measured in urine, such as age, urinary osmolality, and urinary leukocytes. This highlights the importance to take these factors into account when evaluating protein biomarkers in urine. Since urinary pH and osmolality were correlated to some extent in this study group, we did not adjust the final models for urinary pH. A study with Sprague-Dawley rats did not show any effect of urinary pH on urinary proteins determined with urine test strips either (65). Although urinary osmolality is less affected by proteins in urine than other dilution markers such as specific gravity (66), it is not possible to discern the direction of the association between the proteins in this study and osmolality. Storage time of the samples may affect some proteins in plasma (67). In the current study, storage time did not explain the overall protein variation or influenced the top proteins associated with B-As. There were several women with menstrual bleeding, but this did not influence the proteins evaluated. Women with urinary tract infection, identified by the presence of leukocytes in urine, were expected considering the limited access to health care in the study area. However, this did not influence the variation of the proteins.

Regarding the choice of matrices for measuring exposure to arsenic, B-As is most suitable to assess recent exposure to iAs since it is rapidly cleared from this matrix (68). Still, the strong correlation between B-As and U-As concentrations in the current study group likely reflects a chronic exposure to iAs, and it allowed us to use B-As as a valid biomarker of exposure. Arsenic concentrations in drinking water from these villages did not present temporal variations during the 2 years of recruitment, supporting that these women were constantly exposed to iAs (15). By using two different biological matrices to measure exposure (blood) and effect biomarkers (urine), we avoided potential problems of co-excretion in urine, previously identified for other elements like cadmium (29). Furthermore, it would be valuable to analyze these cancer-related proteins in other matrices such as serum or plasma in order to correlate these with urine values, since no data on this is available. Unfortunately, the sampling of plasma from the study individuals was not optimal for protein analysis.

The detection of proteins in urine is commonly considered an indication of kidney disease. High levels of proteins in urine, also known as proteinuria, have been associated with exposure to arsenic (69). In the review by Zheng et al. (69), all studies evaluating proteinuria used U-As as a biomarker of exposure, therefore not being exempt from the potential problem of co-excretion between biomarkers of effect and exposure in the same matrix. The constant development of more advanced protein detection techniques in urine demonstrates that urine is more protein-rich than previously believed, even under normal conditions (70). This, and the fact that urine is an abundant and non-invasive sampling matrix, justifies the attempts to identify more disease biomarkers in urine. In fact, urinary protein biomarkers have been identified as early diagnostic markers for several cancer types (16–18, 71). Regarding the Proximity Extension Assay used in this study, only one other study has employed this technology to evaluate protein expression in urine (72). In Fellström et al. (72), they found an association between lipid markers in serum and inflammation- and cardiovascular-related urinary proteins in 75-year-old individuals, but urinary dilution was not adjusted for in the analyses.

This study group in the Bolivian Andes has a markedly efficient arsenic metabolism capacity (15), which may influence the degree of arsenic toxicity. This, and the fact that cancer types differ between populations depending on their underlying genetic background and susceptibility (73), highlights the need for population-specific studies about arsenic toxicity. This work is a cross-sectional study of apparently healthy women, and therefore we cannot distinguish if the observed variations are adaptive or toxic responses to arsenic exposure. In addition, since iAs is also nephrotoxic (69), it is not possible to disentangle if the associations found are due to arsenic-induced renal toxicity altering protein excretion, or if these proteins serve as proxy for arsenic toxicity in other organs. More research is warranted about the relation between these proteins in different matrices and arsenic-related toxic outcomes for these proteins to be used as toxicity biomarkers or to elucidate the toxicity mechanisms of arsenic. The strengths of this study are the well-characterized arsenic exposure and the use of multiplex proteomic technologies to identify novel candidates to study arsenic-related health effects.

## Conclusion

Using multiplex proteomic methods in urine samples, we identified four putative cancer-related proteins (FASLG, SEZ6L, LYPD3, and TFPI2) associated with arsenic exposure in women living around Lake Poopó, Bolivia. In order to clarify if these proteins represent early arsenic-related carcinogenic changes, follow-up studies are needed. By exploring cancer-related proteins in urine, we hope to contribute to the future development of disease and/or toxicity biomarkers with non-invasive sampling methods.

## Data Availability Statement

The raw data supporting the conclusions of this article will be made available by the authors, without undue reservation.

## Ethics Statement

The studies involving human participants were reviewed and approved by Comité Nacional de Bioética (Bolivia) and the Regional Ethic Committee of Karolinska Institutet (Sweden). The patients/participants provided their written informed consent to participate in this study.

## Author Contributions

JG, NT, and KB planned and designed the research. JD, FA, JG, NT, and KB recruited the study participants and collected the data. ML performed the metal analysis. JD assisted by AG conducted the statistical analyses. JD and KB wrote the manuscript. All authors proofread and commented on previous versions of the manuscript and approved the final version of it.

## Conflict of Interest

The authors declare that the research was conducted in the absence of any commercial or financial relationships that could be construed as a potential conflict of interest.
