# Supplementary material for: Arsenic Exposure and Cancer-Related Proteins in Urine of Indigenous Bolivian Women
Source: Front Public Health. 2020 Dec 14;8:605123. doi: 10.3389/fpubh.2020.605123 (PMC7767847; doi:10.3389/fpubh.2020.605123)
Supplement: Supplementary file 1 [file Presentation_1.PDF]

Supplementary Material

Supplementary Table 1. Operating conditions of ICP-MS (Agilent 7900) for B-As and B-Li determination.

Supplementary Figure 1. Scatter plot of the relation between different arsenic exposure biomarkers. Urinary arsenic (U-As) concentrations presented as the sum of iAs metabolites adjusted to average osmolality. The linear regression line (grey) and loess line (red) are indicated.

Supplementary Table 2. Summary of Normalized Protein eXpression (NPX) values. Data presented as median and interquartile range (IQR) and ordered by descending number of observations above LOD in the total study group (n=176).

Supplementary Figure 2. Heat map of linear models [$-\log_{10}$(*p*-value)] between the principal components explaining the overall variation of protein levels and different covariates. Only proteins with > 40% of observations above LOD were included, and for those, values below LOD were substituted by the protein specific LOD value since the function cannot handle missing data. Numbers on the X-axis of the heat map represent the principal component and in brackets the percentage of variation explained. Lines in the legend key indicate the unadjusted *p*-value threshold (0.05) in blue, and the Bonferroni-adjusted *p*-value threshold (0.05/210) in green to account for multiple comparisons. Figure in color available online.

Supplementary Table 3. Associations between proteins in urine and B-As.

Supplementary Figure 3. Heat map of Spearman correlations between the top proteins associated with B-As (*p*-value < 0.05). Two asterisks indicate *p*-values < 0.01.

Supplementary Table 1. Operating conditions of ICP-MS (Agilent 7900) for B-As and B-Li determination.

Parameter	Setting
Forward plasma power (W)	1550
Sample depth (mm)	5-9
Sampling cone	Ni, 1 mm orifice
Skimmer cone	Ni, 0.4 mm orifice
Spray chamber	Double-pass, quartz
Nebulizer	Mira Mist peek
Torch	Quartz, 2.5 mm id
Nebulizer gas flow (L/min)	0.77-1.00
Dilution gas flow (L/min)	0.25-0.30
Spray chamber cooling temperature (^{o} C)	2
Isotopes monitored	^{7}Li, ^{75}As
Cell gas mode	^{7}Li (no gas), ^{75}As (He 3.0 mL/min)
Internal standards	^{45}Sc, ^{72}Ge, ^{103}Rh, ^{175}Lu, ^{193}Ir
Replicates	4
Sweeps per replicate	100

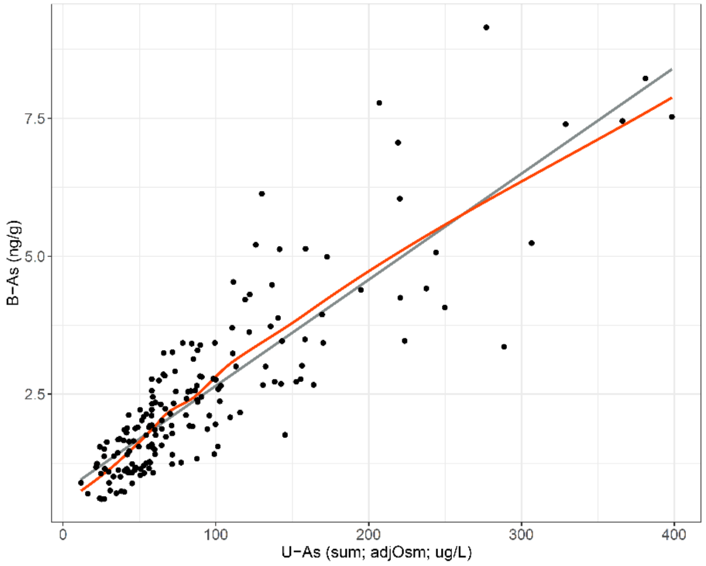

Supplementary Figure 1. Scatter plot of the relation between different arsenic exposure biomarkers. Urinary arsenic (U-As) concentrations presented as the sum of iAs metabolites adjusted to average osmolality. The linear regression line (grey) and loess line (red) are indicated.

Supplementary Table 2. Summary of Normalized Protein eXpression (NPX) values. Data presented as median and interquartile range (IQR) and ordered by descending number of observations above LOD in the total study group (n=176).

HGNC symbol	Olink symbol	n	Median (IQR)
Proteins with > 40% of observations above LOD			
**ADAM8**	ADAM 8	176	3.22 (2.37 - 4.04)
**ANXA1**	ANXA1	176	4.95 (3.51 - 6.25)
**CA9**	CAIX	176	2.74 (1.99 - 3.26)
**CD27**	CD27	176	8.83 (7.72 - 9.49)
**CRNN**	CRNN	176	6.56 (4.58 - 8.64)
**EGF**	EGF	176	11.78 (11.21 - 11.91)
**ERBB3**	ERBB3	176	2.37 (1.83 - 2.79)
**ERBB4**	ERBB4	176	5.9 (5.2 - 6.49)
**FOLR1**	FR-alpha	176	7.11 (5 - 9.36)
**HGF**	HGF	176	5.73 (5.24 - 6.4)
**IFNGR1**	IFN-gamma-R1	176	3.19 (2.57 - 4.07)
**IGF1R**	IGF1R	176	3.13 (2.5 - 3.77)
**SDC1**	SYND1	176	6.57 (5.27 - 7.37)
**TFPI2**	TFPI-2	176	4.89 (4.14 - 5.55)
**WFDC2**	WFDC2	176	9.24 (9.13 - 9.29)
**WISP1**	WISP-1	176	5.27 (4.66 - 5.92)
**CEACAM1**	CEACAM1	174	3.32 (2.8 - 4.1)
**TNFRSF19**	TNFRSF19	174	4.31 (3.4 - 5.16)
**ICOSLG**	ICOSLG	173	2.71 (1.97 - 3.84)
**CD160**	CD160	172	3.99 (2.84 - 5.96)
**PPY**	PPY	170	2.61 (1.84 - 3.5)
**VEGFA**	VEGFA	168	2.86 (1.79 - 4.88)
**LYPD3**	LYPD3	167	2.49 (1.14 - 3.86)
**ITGB5**	ITGB5	160	1.81 (1.51 - 2.2)
**TNFSF13**	TNFSF13	160	2.5 (1.82 - 3.23)
**TGFA**	TGF-alpha	159	0.71 (0.23 - 1.49)
**LY9**	LY9	151	0.7 (0.22 - 1.36)
**SPARC**	SPARC	146	0.48 (0.25 - 0.73)
**FURIN**	FURIN	136	1 (0.28 - 1.94)
**NECTIN4**	PVRL4	133	1.74 (1.08 - 4.05)
**TNFRSF4**	TNFRSF4	131	2.04 (1.66 - 2.74)
**FGFBP1**	FGF-BP1	128	1.67 (1.01 - 2.68)
**KLK11**	hK11	128	0.003 (-1.22 - 1.87)
**MSLN**	MSLN	118	0.12 (-0.12 - 0.54)
**TGFR2**	TGFR-2	105	1.08 (0.89 - 1.59)
**CD70**	CD70	98	1.55 (1.25 - 1.8)
**SEZ6L**	SEZ6L	93	0.63 (0.5 - 0.87)
**KLK13**	KLK13	91	-0.33 (-1.04 - 1.04)
**AREG**	AREG	82	0.71 (0.47 - 1.1)
**ESM1**	ESM-1	81	0.53 (0.35 - 0.79)
**KLK8**	hK8	79	0.13 (-0.75 - 1.11)
**S100A11**	S100A11	76	1.71 (1.28 - 2.63)
**EPHA2**	EPHA2	75	0.65 (0.5 - 1.26)
**FASLG**	FASLG	74	1.44 (1.22 - 2.09)
**CEACAM5**	CEACAM5	73	1.43 (1.11 - 2.4)
Proteins with < 40% observations above LOD - Not included in downstream analyses			
**CXL17**	CXL17	70	0.2 (0.14 - 0.28)
**GPNMB**	GPNMB	68	0.89 (0.75 - 1.3)
**TCL1A**	TCL1A	66	0.74 (0.44 - 1.21)
**METAP2**	MetAP 2	63	0.5 (0.31 - 0.96)
**DLL1**	DLL1	62	1.31 (0.79 - 2.02)
**KDR**	VEGFR-2	60	1.23 (1.02 - 1.63)
**PODXL**	PODXL	59	0.96 (0.84 - 1.05)
**CXCL13**	CXCL13	57	0.94 (0.86 - 1.06)
**FOLR3**	FR-gamma	47	5.01 (2.74 - 6.99)

**XPNPEP2**	XPNPEP2	44	1.27 (1.17 - 1.55)
**KLK14**	hK14	41	-0.46 (-0.87 - 0.25)
**GPC1**	GPC1	38	0.69 (0.53 - 0.96)
**SCAMP3**	SCAMP3	35	1.71 (1.58 - 2.05)
**KITLG**	SCF	33	0.61 (0.43 - 0.79)
**ITGAV**	ITGAV	32	0.41 (0.38 - 0.47)
**TLR3**	TLR3	31	0.44 (0.22 - 0.84)
**ADAMTS15**	ADAM-TS 15	30	0.86 (0.79 - 0.92)
**CYR61**	CYR61	28	0.52 (0.48 - 0.6)
**MDK**	MK	28	0.52 (0.44 - 0.6)
**S100A4**	S100A4	25	1.68 (1.45 - 2.63)
**CPE**	CPE	23	1.27 (0.98 - 1.76)
**TNFRSF6B**	TNFRSF6B	21	0.73 (0.7 - 0.78)
**ABL1**	ABL1	16	0.73 (0.6 - 0.92)
**CDKN1A**	CDKN1A	11	0.49 (0.46 - 0.55)
**CD207**	CD207	9	0.11 (0.04 - 0.22)
**CD48**	CD48	9	1.4 (1.32 - 2.35)
**MUC16**	MUC-16	9	1.6 (1.36 - 2.95)
**LGALS1**	Gal-1	8	0.78 (0.63 - 1.46)
**MIC-A/B**	MIC-A/B	8	0.42 (0.4 - 0.51)
**CTSV**	CTSV	7	-0.92 (-1.11 - -0.36)
**FLT4**	VEGFR-3	7	0.85 (0.83 - 0.88)
**IL6**	IL6	7	1.09 (0.97 - 1.16)
**GZMH**	GZMH	6	0.52 (0.45 - 0.6)
**RET**	RET	6	0.14 (0.12 - 0.16)
**TNFSF10**	TRAIL	6	1.72 (1.63 - 2.12)
**RSPO3**	RSPO3	4	0.58 (0.57 - 0.6)
**ERBB2**	ERBB2	3	-0.03 (-0.06 - -0.02)
**FADD**	FADD	3	1.03 (1.02 - 1.11)
**FCRLB**	FCRLB	3	0.43 (0.42 - 0.44)
**LYN**	LYN	3	1.16 (0.83 - 1.32)
**SMAD5**	MAD homolog 5	3	0.75 (0.63 - 1.62)
**TXLNA**	TXLNA	2	0.87 (0.87 - 0.88)
**WIF1**	WIF-1	2	0.92 (0.85 - 1)
**VIM**	VIM	2	3.18 (2.64 - 3.72)
**MIA**	MIA	1	0.54 (0.54 - 0.54)
**NT5E**	5-NT	1	1.19 (1.19 - 1.19)
**GZMB**	GZMB	0	NA

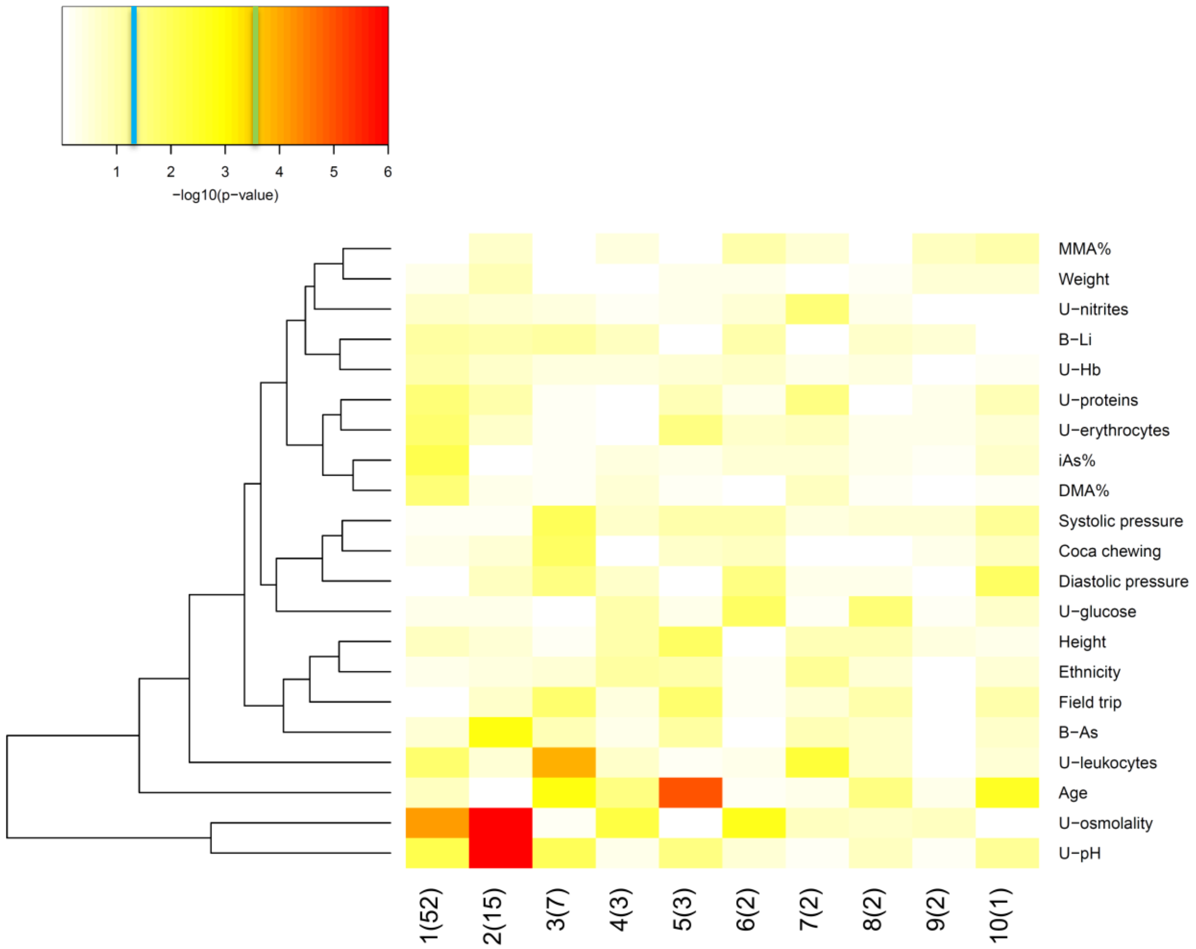

Supplementary Figure 2. Heat map of linear models [-$\log_{10}$(p-value)] between the principal components explaining the overall variation of protein levels and different covariates. Only proteins with > 40% of observations above LOD were included, and for those, values below LOD were substituted by the protein specific LOD value since the function cannot handle missing data. Numbers on the X-axis of the heat map represent the principal component and in brackets the percentage of variation explained. Lines in the legend key indicate the unadjusted p-value threshold (0.05) in blue, and the Bonferroni-adjusted p-value threshold (0.05/210) in green to account for multiple comparisons. Figure in color available online.

Abbreviations and units: Age (years); B-As, whole blood arsenic (ng/g); B-Li, whole blood lithium (ng/g); Coca chewing (yes/no); DMA, dimethylarsinic acid; Ethnicity (Aymara-Quechua or Uru); iAs, inorganic arsenic; MMA, monomethylarsonic acid; Systolic and diastolic blood pressure (mmHg); U-, indicates characteristic measured in urine; U-Hb, urinary hemoglobin; U-osmolality (mOsm/kg); and Weight (kg).

Supplementary Table 3. Associations between proteins in urine and B-As.

	Main analysis [a]				Sensitivity analysis [b]			
**Protein**	**n**	**R^2 (%)**	**B (95% CI)**	**p-value**	**n**	**R^2 (%)**	**B (95% CI)**	**p-value**
**FASLG**	74	5	0.155 (0.034, 0.276)	0.013	68	4	0.165 (-0.001, 0.331)	0.052
**SEZ6L**	93	25	-0.038 (-0.072, -0.004)	0.028	86	24	-0.044 (-0.091, 0.003)	0.067
**LYPD3**	166	23	-0.172 (-0.331, -0.014)	0.034	150	22	-0.189 (-0.381, 0.003)	0.054
**TFPI2**	175	24	-0.125 (-0.249, -0.001)	0.049	159	24	-0.117 (-0.27, 0.036)	0.132
**AREG**	82	15	-0.096 (-0.204, 0.012)	0.080	79	16	-0.074 (-0.186, 0.039)	0.195
**CA9**	175	49	-0.05 (-0.112, 0.013)	0.119	159	48	-0.036 (-0.113, 0.04)	0.352
**ESM1**	81	7	-0.067 (-0.152, 0.019)	0.124	74	5	-0.041 (-0.145, 0.063)	0.435
**ICOSLG**	172	3	-0.097 (-0.225, 0.03)	0.134	156	3	-0.044 (-0.199, 0.111)	0.576
**CD70**	98	11	-0.046 (-0.106, 0.015)	0.137	90	12	-0.012 (-0.089, 0.065)	0.765
**TNFRSF4**	131	27	-0.066 (-0.164, 0.032)	0.183	122	28	-0.043 (-0.158, 0.072)	0.457
**TNFSF13**	159	29	-0.066 (-0.17, 0.038)	0.213	144	27	-0.052 (-0.185, 0.081)	0.441
**FOLR1**	175	-1	0.14 (-0.11, 0.389)	0.271	159	-1	0.244 (-0.062, 0.55)	0.117
**KLK11**	127	0	0.091 (-0.119, 0.301)	0.391	117	0	0.153 (-0.097, 0.403)	0.229
**KLK13**	91	-1	0.081 (-0.123, 0.284)	0.434	86	4	0.255 (0.002, 0.508)	0.048
**SPARC**	145	18	-0.014 (-0.05, 0.022)	0.452	130	17	-0.02 (-0.068, 0.029)	0.425
**ADAM8**	175	27	-0.041 (-0.148, 0.066)	0.453	159	27	-0.028 (-0.156, 0.101)	0.673
**ITGB5**	160	27	-0.018 (-0.066, 0.03)	0.467	144	27	-0.015 (-0.073, 0.043)	0.615
**TGFA**	158	20	-0.028 (-0.107, 0.05)	0.473	142	22	-0.012 (-0.107, 0.083)	0.807
**TGFR2**	105	14	-0.03 (-0.117, 0.056)	0.490	96	15	-0.078 (-0.194, 0.038)	0.186
**TNFRSF19**	173	29	-0.036 (-0.146, 0.074)	0.521	157	31	-0.022 (-0.152, 0.108)	0.740
**CEACAM5**	72	4	-0.054 (-0.235, 0.126)	0.552	67	2	-0.031 (-0.232, 0.17)	0.757
**KLK8**	79	2	-0.046 (-0.215, 0.124)	0.592	75	1	-0.006 (-0.205, 0.193)	0.955
**ANXA1**	175	7	-0.048 (-0.227, 0.131)	0.594	159	5	-0.037 (-0.256, 0.181)	0.735
**WFDC2**	175	37	-0.006 (-0.027, 0.016)	0.605	159	38	-0.015 (-0.042, 0.012)	0.273
**IGF1R**	175	29	-0.021 (-0.104, 0.062)	0.620	159	31	-0.013 (-0.113, 0.088)	0.804
**EPHA2**	75	-3	0.021 (-0.073, 0.115)	0.653	70	-2	0.054 (-0.061, 0.169)	0.354
**WISP1**	175	50	-0.018 (-0.101, 0.064)	0.659	159	51	-0.021 (-0.123, 0.08)	0.678
**S100A11**	76	5	-0.033 (-0.201, 0.135)	0.696	73	6	0.001 (-0.191, 0.194)	0.990
**HGF**	175	33	-0.016 (-0.101, 0.07)	0.718	159	34	-0.023 (-0.128, 0.083)	0.669
**FGFBP1**	128	2	-0.025 (-0.165, 0.115)	0.724	120	3	0.016 (-0.146, 0.179)	0.842
**NECTIN4**	132	-1	0.034 (-0.16, 0.228)	0.730	121	-2	0.086 (-0.159, 0.33)	0.490
**CRNN**	175	9	-0.041 (-0.275, 0.193)	0.731	159	8	0.035 (-0.25, 0.321)	0.806
**LY9**	151	14	-0.013 (-0.091, 0.065)	0.738	138	16	0.026 (-0.069, 0.122)	0.587
**FURIN**	136	1	-0.021 (-0.148, 0.105)	0.741	124	0	0.011 (-0.144, 0.165)	0.892
**PPY**	170	34	-0.018 (-0.132, 0.096)	0.755	154	37	-0.079 (-0.211, 0.054)	0.242
**SDC1**	175	37	-0.018 (-0.133, 0.097)	0.756	159	37	-0.053 (-0.194, 0.087)	0.455
**ERBB3**	175	37	-0.008 (-0.067, 0.051)	0.781	159	37	0 (-0.073, 0.072)	0.994
**CEACAM1**	173	14	-0.011 (-0.097, 0.074)	0.790	157	13	0.01 (-0.094, 0.113)	0.857
**CD27**	175	14	-0.018 (-0.16, 0.123)	0.799	159	18	0.029 (-0.134, 0.192)	0.728
**CD160**	171	10	-0.023 (-0.21, 0.163)	0.807	155	9	-0.011 (-0.24, 0.218)	0.925
**EGF**	175	20	0.011 (-0.099, 0.122)	0.838	159	20	0.017 (-0.12, 0.154)	0.808
**MSLN**	118	7	-0.009 (-0.11, 0.093)	0.868	108	5	-0.011 (-0.153, 0.13)	0.873
**VEGFA**	167	-2	0.013 (-0.186, 0.212)	0.898	153	-3	0.093 (-0.15, 0.335)	0.452
**ERBB4**	175	22	-0.002 (-0.102, 0.097)	0.963	159	22	0.02 (-0.103, 0.142)	0.753
**IFNGR1**	175	19	0.001 (-0.098, 0.1)	0.981	159	19	0.037 (-0.084, 0.158)	0.547

Data presented as B coefficients and 95% confidence intervals (CI) for the association with B-As.
No proteins passed the Bonferroni adjustment for multiple comparison (*p*-value 0.05/45).
Analyses restricted to those proteins with > 40% observations above LOD.
[a] Multivariate linear regression models adjusted for age, U-osmolality, and U-leukocytes.
[b] Multivariate linear regression models adjusted for age, U-osmolality, U-leukocytes, and B-Li. The number of individuals differs from the main analysis because some individuals did not have B-Li data available.

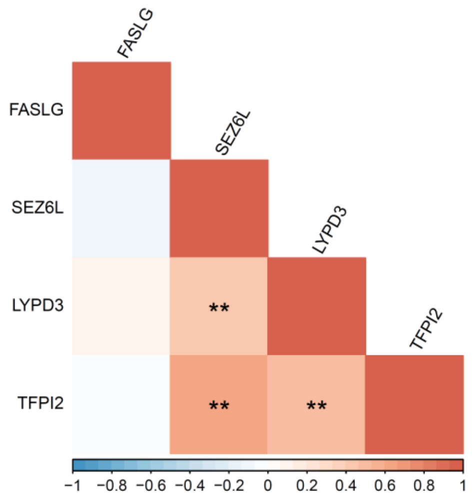

Supplementary Figure 3. Heat map of Spearman correlations between the top proteins associated with B-As (p-value < 0.05). Two asterisks indicate p-values < 0.01.
